# Pressure from 2D snapshot PIV

**DOI:** 10.1007/s00348-019-2678-5

**Published:** 2019-01-25

**Authors:** J. W. Van der Kindere, A. Laskari, B. Ganapathisubramani, R. de Kat

**Affiliations:** 1 Department of Aeronautics and Astronautics, University of Southampton, Southampton, SO17 1BJ UK; 2 Present Address: GALCIT, California Institute of Technology, Pasadena, CA 91125 USA

## Abstract

**Abstract:**

In this study, we quantify the accuracy of a simple pressure estimation method from 2D snapshot PIV in attached and separated flows. Particle image velocimetry (PIV) offers the possibility to acquire a field of pressure instead of point measurements. Multiple methods may be used to obtain pressure from PIV measurements, however, the current state-of-the-art requires expensive equipment and data processing. As an alternative, we aim to quantify the efficacy of estimating instantaneous pressure from snapshot (non-time resolved) two-dimensional planar PIV (the simplest type of PIV available). To make up for the loss of temporal information, we rely on Taylor’s hypothesis (TH) to replace temporal information with spatial gradients. Application of our approach to high-resolution 2D velocity data of a turbulent boundary layer flow over ribs shows moderate to good agreement with reference pressure measurements in average and fluctuations. To assess the performance of the 2D TH method beyond average and fluctuation statistics, we acquired a time-resolved measurement of the same flow and determined temporal correlation values of the pressure from our method with reference measurements. Overall, the correlation attains good values for all measured locations. For comparison, we also applied two time-resolved approaches, which attained values of correlation similar to our approach. The performance of the 2D TH method is further assessed on 3D time-resolved velocity data for a turbulent boundary layer and compared with 3D methods. The root-mean-square (RMS) pressure fluctuations of the 2D TH, 3D TH and 3D pseudo-Lagrangian methods closely follow the pressure fluctuation distribution from DNS. These observations on the RMS pressure estimates are further supported by similar analysis on synthetic PIV data (based on DNS) of a turbulent channel flow. The values of spatial correlation between the 2D TH method and the DNS pressure fields in this case, are similar to the temporal correlations achieved in the turbulent flow over the ribs. Finally, we discuss the accuracy of instantaneous pressure estimates and provide a rule of thumb to determine regions where the pressure fluctuation estimate from the 2D TH methods is likely to fail.

**Graphical abstract:**

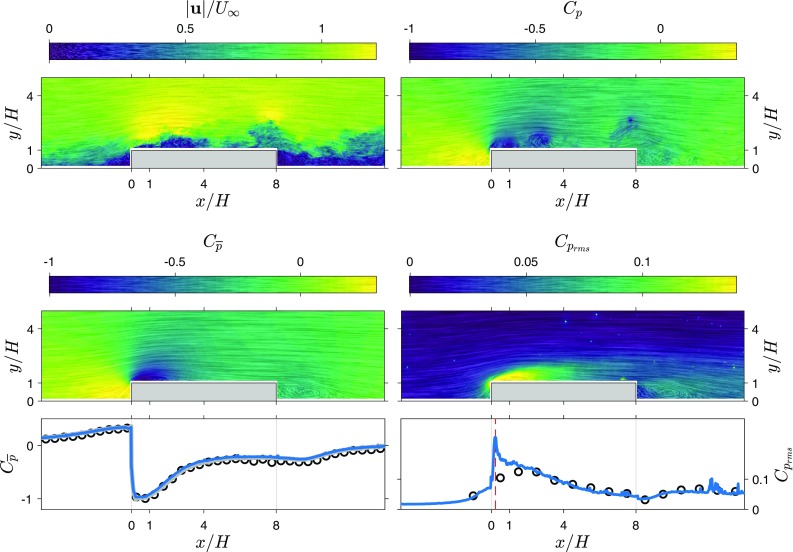

**Electronic supplementary material:**

The online version of this article (10.1007/s00348-019-2678-5) contains supplementary material, which is available to authorized users.

## Introduction

In recent years, the rapid development of particle image velocimetry (PIV) and post-processing techniques has led to a fast increase in temporal and spatial resolution of velocity data (Scarano 2013) that currently allows for full-field pressure estimation (van Oudheusden 2013). In contrast, conventional point-wise pressure measurements, crucial to a variety of industrial applications, are limited in the information they provide and in the potential for improvement. Therefore, there is a great interest in techniques estimating pressure from flow velocity information. These techniques generally use the Navier–Stokes equations, where all velocity terms can be measured directly through PIV measurements, and solve for the remaining pressure term.

Starting with the work of Gurka et al. (1999) when time-resolved data were not yet readily accessible, planar PIV velocity snapshots of a pipe and jet flow were used to get time-averaged pressure using a Poisson formulation of the Reynolds-averaged Navier–Stokes (RANS) equations and results were compared with data from previous studies. In a similar line of work, Hosokawa et al. (2003), used both planar PIV and particle tracking velocimetry (PTV) data of a laminar liquid flow around bubbles, to estimate time-averaged pressure using an iterative Poisson solver. Apart from a Poisson formulation, alternative schemes of pressure integration were also analysed, most notably in the work by Liu and Katz (2006) who employed an omni-directional virtual integration scheme to get pressure in a cavity shear flow. Later, van Oudheusden et al. (2007) also evaluated time-averaged pressure and forces from planar PIV data in both compressible and incompressible flow cases using a control volume approach and a spatial integration scheme for the pressure gradients.

Building on these time-averaged studies, Fujisawa et al. (2005) were among the first to attempt instantaneous pressure determination, using snapshot PIV on the flow around a cylinder, although the missing time information posed specific constraints in the boundary conditions used. As high-speed PIV systems started to develop, time-resolved velocity information became available and several studies on instantaneous pressure determination subsequently emerged (Liu and Katz 2006; Murai et al. 2007; de Kat et al. 2008), effectively shifting the attention towards evaluation methods for the material acceleration term. In that context both Eulerian and Lagrangian approaches for the computation of the acceleration term were assessed in various flow scenarios, often with contradicting results (see Jakobsen et al. 1997; Charonko et al. 2010; Violato et al. 2011; de Kat and van Oudheusden 2012; Ghaemi et al. 2012, among others). An Eulerian approach was found to better match experimental results in the case of surface waves (Jakobsen et al. 1997), while a Lagrangian method was shown to be limited due to poor particle tracking and exhibited a small bias leading to a systematic error in pressure estimation. These results were supported by de Kat and van Oudheusden (2012) who attributed the limitations of a pseudo-Lagrangian technique—pseudo, because fluid parcel paths are estimated from the velocity data instead of actually tracking a fluid parcel or tracer particle—due to the structures’ turnover time, to be more severe than the ones for an Eulerian approach. In contrast to this, works from both Violato et al. (2011) and Ghaemi et al. (2012) showed that a pseudo-Lagrangian approach managed a lower precision error and was less sensitive to noise in comparison with an Eulerian method.

However, new techniques that allow for accurate fully Lagrangian particle tracking have recently been developed and show great promise in improving material acceleration measurements (and therefore pressure estimation). Most notably, Schröder et al. (2015), and Schanz et al. (2016) presented a ‘Shake the Box’ algorithm, which uses volumetric time-resolved particle images and reconstructs particle trajectories using previous time steps to predict future particle positions and corrects accordingly using image matching. Such particle tracking methods provide highly accurate material acceleration estimations, significantly improving pressure reconstruction when compared to methods that use PIV velocity information, which is known to suffer from averaging effects of the cross-correlation process involved (see van Gent et al. 2017, for a detailed comparison on pressure estimation using different PIV and PTV based approaches).

These developments show that in a scientific context, progress on pressure estimation techniques is oriented towards state-of-the-art equipment and complex computational algorithms. However, for some applications (e.g. industrial measurements), minimisation of cost, complexity and processing time is critical, and often balanced against the accuracy of the technique. Therefore, to allow an informed choice in this balance, it is valuable to quantify how loss of information (either in terms of time or space) affects the accuracy of pressure estimation methods, so that a balance between cost and performance can be found for different applications (see, e.g., McClure and Yarusevych 2017, for an exposition of 2D vs 3D measurements, time and spatial resolution in turbulent wake flows).

In a bid to simplify the measurement equipment needed, one could use models to remove the requirement to capture a time-series altogether. Schneiders et al. (2016) proposed a method based on the vorticity transport equation to estimate instantaneous pressure from 3D velocity snapshots when time information is not available, however, this is a complex procedure which still requires (complicated) volumetric-PIV/PTV measurement.

A different approach is to use Taylor’s hypothesis (TH) to fill in the missing spatial information (de Kat and Ganapathisubramani 2013) or time information (de Kat and Ganapathisubramani 2013; Laskari et al. 2016). The use of TH was assessed in the case of time-resolved 3C-planar data (de Kat and Ganapathisubramani 2013) and 3C-volumetric snapshots (Laskari et al. 2016) and reliable pressure estimates were found for both, missing spatial and temporal information respectively, while the TH method also proved to be the most robust approach with respect to noise and grid resolution (Laskari et al. 2016).

However, these studies still require either time-resolved stereo or snapshot tomographic PIV to acquire 3C velocity data. Our goal in the present work is to assess the performance of the TH approach in the simplest possible setup, snapshot 2D PIV.

We assess the performance of pressure determination with the TH method using 2D velocity snapshots in both separated and wall-bounded flows and compare its performance versus reference measures. Additionally—measurement data allowing—we provide comparisons with alternative techniques. We start with describing the different approaches used in this study, before applying them to four different data sets, with the main goal to assess different performance aspects of the 2D TH method.

First, we showcase pressure estimation using high-resolution 2D snapshot measurements, where no other technique applies, and consider ribs of different lengths which allow to assess the influence of size (and strength) of the separation region (Van der Kindere and Ganapathisubramani 2018). The resulting estimated pressures are compared statistically with wall pressure measurements.

Second, because statistical measures can obscure and balance noise sources, we use 2D time-resolved data to determine the temporal cross-correlation of the pressure signal with wall pressure measurements, which provides information on how closely the estimated and measured time-series match in time. These time-resolved data also allow for 2D time-resolved pressure estimation approaches, therefore, for completeness, their results are included.

Third, because turbulent flows are inherently 3D we will apply the 2D TH method to the data from Laskari et al. (2016): 3D-3C experimental data of a turbulent boundary layer and a DNS-based synthetic PIV data of a channel flow. Following the same analysis, this allows us to assess the impact on the results when going from a 3D TH method to a 2D TH method, both in an experimental (boundary layer) case and a synthetic PIV (channel) case, where the latter also allows for a comparison with the ground truth pressure from DNS. For completeness, results from time-resolved 3D approaches are included.

The results of the current study will be discussed and compared with the results of the snapshot pressure estimation approach by Schneiders et al. (2016), the accuracy of instantaneous pressure estimates and root-mean-square (RMS) pressure estimate will be presented, and a rule-of-thumb will be discussed, before the main conclusions of the work are summarised.

## Pressure estimation from PIV data

Before the assessment of their performance, the different approaches for estimating pressure from PIV used in this manuscript are briefly introduced here.

The main difference between the approaches lies in the way the pressure gradients are determined, which depends on the available velocity data. After the velocity data have been acquired, pressure gradients can be estimated from velocity data using the Navier–Stokes equation:

1 $$\begin{aligned} \nabla p=-\rho \left\{ \frac{\partial \mathbf {u}}{\partial t}+\left( \mathbf {u}\cdot \nabla \right) \mathbf {u}-\nu \nabla ^{2}\mathbf {u}\right\} , \end{aligned}$$

where the terms on the right hand side can be determined in different ways—depending on available data.

The Reynolds-averaged Navier–Stokes equation (RANS, see, e.g., van Oudheusden et al. 2007) can be used to estimate the average pressure gradient from PIV statistics. Reynolds averaging ($\mathbf {u}=\overline{\mathbf {u}}+\mathbf {u}'$, where $\mathbf {u}$ is the instantaneous velocity, $\overline{\mathbf {u}}$ is the time (or ensemble) averaged velocity, and $\mathbf {u}'$ the velocity fluctuation around the average) is applied to the (incompressible) Navier–Stokes equation, and yields time-averaged pressure gradients (see, e.g., van Oudheusden et al. 2007) which only depend on the statistics of PIV measurements:

2 $$\begin{aligned} \nabla {\overline{p}} = -\rho \{ (\overline{\mathbf {u}} \cdot \nabla )\overline{\mathbf {u}}+ \nabla \cdot \overline{\mathbf {u}'\mathbf {u}'} - \nu \nabla ^2 \overline{\mathbf {u}} \}, \end{aligned}$$

where the velocity gradients can be determined using a central difference scheme:

3 $$\begin{aligned} \frac{\partial \mathbf {u}}{\partial x}\left( \mathbf {x},t\right) =\frac{\mathbf {u}\left( \mathbf {x}+h\mathbf {e}_x,t\right) -\mathbf {u}\left( \mathbf {x}-h\mathbf {e}_x,t\right) }{2h}, \end{aligned}$$

where *h* is the grid spacing. This approach will be applied in Sect. 3.

An Eulerian approach (EU, based on de Kat and van Oudheusden 2012) uses the instantaneous momentum balance, see Eq. 1, and requires time information. The spatial velocity gradients and the local acceleration can be determined using a central difference scheme, using Eq. 3 and

4 $$\begin{aligned} \frac{\partial \mathbf {u}}{\partial t}\left( \mathbf {x},t\right) =\frac{\mathbf {u}\left( \mathbf {x},t+ {\varDelta }t\right) -\mathbf {u}\left( \mathbf {x},t- {\varDelta }t\right) }{2{\varDelta } t}, \end{aligned}$$

respectively, where ${\varDelta }t$ is the time separation between consecutive velocity fields. This approach is applied in Sects. 4 and 5.

A pseudo-Lagrangian approach (pLA, based on de Kat and van Oudheusden 2012) also uses the instantaneous momentum balance to estimate the instantaneous pressure gradient, but instead of determining the spatial and temporal components individually, the material derivative is directly estimated:

5 $$\begin{aligned} \nabla p=-\rho \left\{ \frac{\mathrm {D}\mathbf {u}}{\mathrm {D} t}-\nu \nabla ^{2}\mathbf {u}\right\} , \end{aligned}$$

and therefore also requires time information. For this pseudo-Lagrangian approach, an iterative second order particle path fit is used to estimate the material derivative:

6 $$\mathbf {x}_p^k\left( t,\tau \right) = \mathbf {x}+\mathbf {u}\left( \mathbf {x},t\right) \tau + \frac{1}{2}\frac{\mathrm {D} \mathbf {u}}{\mathrm {D}t}^{k}\left( \mathbf {x},t\right) \tau ^2$$

7 $$\frac{\mathrm {D} \mathbf {u}}{\mathrm {D}t}^{k+1}\left( \mathbf {x},t\right) = \frac{\mathbf {u}\left( \mathbf {x}_p^k\left( t, {\varDelta } t\right) ,t+ {\varDelta } t \right) -\mathbf {u}\left( \mathbf {x}_p^k\left( t,- {\varDelta } t\right) ,t- {\varDelta } t \right) }{2 {\varDelta } t}.$$

This approach is applied in Sects. 4 and 5.

A Taylor’s hypothesis approach (TH, based on de Kat and Ganapathisubramani 2013; Laskari et al. 2016) can be used to estimate the local acceleration needed for instantaneous pressure gradient estimation without the need of time information. Taylor’s hypothesis (Taylor 1938) uses Reynolds averaging and the assumption that the fluctuations are frozen and move with the mean velocity:

8 $$\begin{aligned} \frac{\partial \mathbf {u}'}{ \partial t} =- (\overline{\mathbf {u}} \cdot \nabla ) \mathbf {u}' . \end{aligned}$$

For Taylor’s hypothesis to hold, the fluctuations need to be sufficiently small with respect to the local mean velocity. For strongly turbulent and separated flows this is generally not the case, but—as de Kat and Ganapathisubramani (2013) and Laskari et al. (2016) showed—errors in convection velocity can be less detrimental than the noise associated with the direct determination of the local acceleration (or material acceleration).

The use of an appropriate convection velocity in the Taylor’s hypothesis formulation has been the subject of many studies. Using the mean as the convection velocity was shown to be appropriate in the case of grid generated decaying turbulence (Favre et al. 1955), but not in cases where shear is present (Lin 1953), in which the fluctuations are convected with the local mean velocity only in limited regions of the flow and the hypothesis would break down elsewhere (Fisher and Davies 1964; Zaman and Hussain 1981; Kim and Hussain 1993; Davoust and Jacquin 2011, among others). de Kat and Ganapathisubramani (2013) showed that using an in-plane filtered axial velocity in a turbulent jet as the convection velocity, yields more promising results for pressure, than using the mean. More recently, Geng et al. (2015) tested the validity of using the mean as the convection velocity in the case of a turbulent channel and concluded that the assumption holds well in the logarithmic and outer layer but fails close to the wall, where the convection velocity does not tend to zero as the local mean velocity, but reaches a constant (non-zero) value. Also for wall-bounded flows, Laskari et al. (2016) and van Gent et al. (2017) found good results using the local mean velocity (for a channel flow and turbulent boundary layer, and a backward facing step, respectively). Therefore, in this study, we will use the mean velocity as an estimate for the convection velocity.

Since the mean velocity is not a function of time, we can replace the local acceleration in Eq. 1 with the convection term from Eq. 8 and the instantaneous pressure gradient then is only a function of spatial velocity information:

9 $$\begin{aligned} \nabla p = - \rho \{ -(\overline{\mathbf {u}} \cdot \nabla ) \mathbf {u}' + (\mathbf {u}\cdot \nabla ) \mathbf {u} - \nu \nabla ^2 \mathbf {u} \}. \end{aligned}$$

This approach is applied in Sects. 3, 4 and 5.

### Integration into pressure fields

Now that we have estimates for the pressure gradient, the divergence of these estimates is determined and the resulting Poisson formulation can be integrated using a Poisson solver (see e.g. de Kat and van Oudheusden 2012). For example, for the RANS approach, this results in the following Poisson formulation:

10 $$\begin{aligned} \nabla ^2 \overline{p} = \nabla \cdot \left( -\rho \left\{ ( \overline{\mathbf {u}} \cdot \nabla ) \overline{\mathbf {u}} + \nabla \cdot \overline{\mathbf {u}'\mathbf {u}'} - \nu \nabla ^2 \overline{\mathbf {u}} \right\} \right) . \end{aligned}$$

And for the TH approach this results in the following Poisson formulation:

11 $$\begin{aligned} \nabla ^2 p = \nabla \cdot \left( - \rho \left\{ -(\overline{\mathbf {u}} \cdot \nabla ) \mathbf {u}' + (\mathbf {u}\cdot \nabla ) \mathbf {u} - \nu \nabla ^2 \mathbf {u} \right\} \right) . \end{aligned}$$

To have a well-posed problem, we need to apply some boundary conditions in addition to Eqs. 10 and 11. In this study, the region of integration is bounded by a Dirichlet boundary condition along the top, imposing a pressure determined by a modified Bernoulli’s equation (see de Kat and van Oudheusden 2012), and three Neumann boundary conditions at the upstream, downstream and wall surface boundaries of the domain. For determining the Dirichlet pressure values, free stream pressure is used as a reference. This reference pressure was measured by Pitot-static tube during the PIV data acquisition. This approach to boundary conditions is a common configuration in wall-bounded flows (see, e.g., de Kat and van Oudheusden 2012; Ghaemi et al. 2012; Laskari et al. 2016; van Gent et al. 2017).

### 2D flow assumption

Generally, turbulent flows are three-dimensional. However, planar PIV only measures the in-plane velocity components and in-plane gradients. Therefore, only a 2D version (i.e.,  without out-of-plane velocity gradients, and therefore out-of-plane velocity components) of the pressure estimation techniques can be employed. The introduced error has previously been quantified using synthetic vortex flows (see Charonko et al. 2010; de Kat and van Oudheusden 2012), where de Kat and van Oudheusden showed that the pressure deviated with the cosine of the vortex angle with respect to the plane normal. In fully homogeneous turbulent flow, vortices point in random directions, therefore a loss of pressure signal of more than 50% is expected for the 2D flow assumption.

However, in certain cases, the impact of this error on the pressure signal is limited. The impact of the three-dimensionality on the pressure signal was investigated in more detail by McClure and Yarusevych (2017), who found that the error due to the 2D flow assumption increases with Reynolds number, but remained limited to 5% for the pressure in the wake of a circular cylinder at $Re_\mathrm{D}=1600$. For a higher Reynolds number, $Re_\mathrm{D}=$ 10,000, square cylinder flow, (de Kat and van Oudheusden 2012) found a difference of about 5% in RMS response and about 10% difference between peak correlation for 2D and 3D approaches.

Therefore, it appears that the error due to the 2D flow assumption might not always be as severe as expected (much smaller than 50%) and is therefore worthwhile investigating.

## Pressure estimation from snapshot planar PIV data and comparison with wall pressure statistics

In this section, the TH method is applied to snapshot planar PIV data and the resulting pressure statistics are compared to direct pressure measurements. Mean pressure is also estimated using the RANS method (described in Sect. 2) for further comparison. The performance of the method is investigated in a series of separating and reattaching flows where the extent of separation behind ribs is varied by varying the length to height ratio of the rib ($L/H=1$, 4, and 8, for the influence of the rib length on the separation region see e.g. Van der Kindere and Ganapathisubramani 2018).

**Fig. 1:**
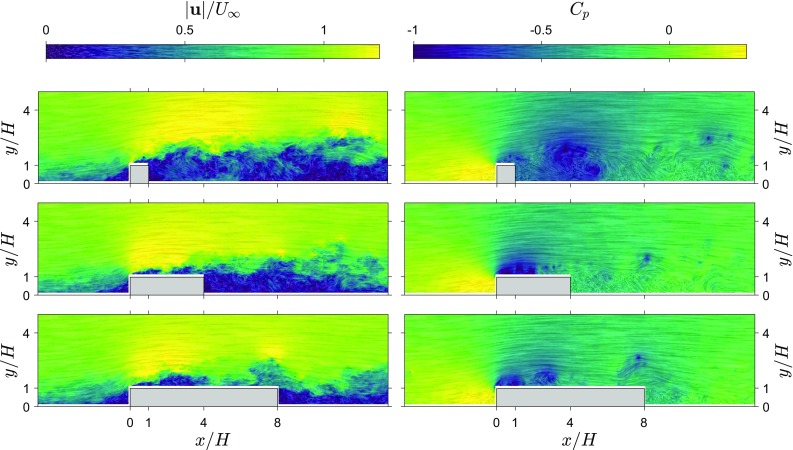
High resolution snapshot velocity magnitude and snapshot pressure contours for three rib lengths ($L/H=1,4$ and 8). Line integral convolution indicates local streamlines and strength. Left: instantaneous velocity magnitude fields, $|\mathbf {u}|/U_\infty$. Right: instantaneous pressure fields, $C_\mathrm{p}$

For this kind of snapshot planar PIV data, time information is not available and therefore the only available technique to estimate pressure from the PIV data is the 2D TH method.

### Experimental set-up of ribs of varying length

To obtain the wall pressure measurements and snapshot 2D PIV, we carried out experiments in the “3 $\times$ 2” wind-tunnel at the University of Southampton where a high-resolution planar PIV was used to capture velocity snapshots and surface tappings and pressure transducers were used to capture mean and fluctuation wall pressure. Rib height *H*, was kept constant while the streamwise aspect ratio of the rib was varied ($L/H=1,4,8$) to assess its influence on the flow separation region (see Bergeles and Athanassiadis 1983, for a detailed description of rib length influence on the flow separation in a turbulent boundary layer).

The PIV equipment included two Litron Nano L 200-15 lasers, three LaVision LX Imager 16 MP PIV cameras with Sigma 105 mm F/2.8 Macro lenses, and a set of Thorlabs laser optics for beam alignment. Each individual element was necessary only once for the simplest form of PIV, however, multiple of each were required to capture the large field of view ($-5.4 \le x/H \le 14.5$, and $0 \le y/H \le 6$ with $x/H=0, y/H=0$ at the bottom of the upstream face of the rib) and avoid occlusion in the region in front of and behind the rib.

For each rib length, 2000 particle image pairs were acquired with Davis 8.1 (at 0.2 Hz), which were then processed using an iterative triple cross-correlation procedure with a decreasing window size (from 64 $\times$ 64 to 16 $\times$ 16 pixels, with 50% overlap). The image pairs were pre-processed using background subtraction, while the resulting velocity fields were post-processed by removing and replacing outliers outside three times the local standard deviation. The PIV configuration allowed a final vector spacing of $\approx 0.5$ mm corresponding to $\approx 60$ vectors per rib height ($H/h=60$). The uncertainty due to correlation noise on the resulting velocity was estimated to be 2% based on the minimum measurable turbulence intensity. To assess the influence of spatial resolution, the velocity data were filtered with a moving average and downsampled ($H/h=30$, $H/h=20$, $H/h=15$, $H/h=10$, and $H/h=7.5$) to mimic lower resolution PIV setups.

Two separate systems were used for surface pressure measurements. The first was a Scanivalve ZOC 22B with up to 48 channels for mean pressure measurements. Mean pressure was acquired through a series of 0.6 mm circular taps, mounted flush with the surface, with a streamwise spacing of 0.5*H*, the first one located at $x/H= -8.25$. The taps were aligned in the streamwise direction with the PIV measurement plane. The second system was necessary for pressure fluctuations. It consisted of two Endevco 8507C-2 pressure transducers and a third Endevco 8510-1B connected to the surface by a 0.8 mm wide, 2 mm long hole. The 8507C-2 transducers measured pressure fluctuations from $x/H=-1$ to 14.5, whereas the 8510-1B transducer was placed far upstream to capture uncorrelated pressure signal that is necessary for optimal noise cancellation as per Naguib et al. (1996). In total, the transducers were sampled four times for 30 s at the rate of 25.6 kHz. The signal from each transducer was filtered first through optimal noise removal filter and subsequently low-pass filtered at 1280 Hz.

### Instantaneous snapshot pressure results

In keeping with a simple PIV configuration, only uncorrelated snapshot PIV measurements were obtained in this set of experiments. This allows the use of the 2D TH method to obtain instantaneous pressure from the snapshot data and subsequently, pressure statistics.

Figure 1 shows a sample of the original velocity distribution around three ribs ($L/H=1,4$ and 8) in the form of line integral convolution (LIC) as well as the corresponding instantaneous pressure distribution obtained using the 2D TH method. The line integral convolution was inspired by Phillips et al. (2015) and Longmire et al. (2003), and was implemented in-house as per the description of Cabral and Leedom (1993) using the multi-frequency noise technique described by Kiu and Banks (1996). The combination of these techniques provides the clearest visualisation of flow trajectory in our PIV snapshots with indications of coherent regions such as vortices shed from the obstacles.

Qualitatively, known phenomena are well represented in the pressure field. Most notably, points of high vorticity near the leading edge of the ribs correspond to vortices being shed at this location and travelling downstream along the shear layer. This phenomenon appears in the form of trains of low pressure pulses, matching the centre of each vortex. Furthermore, in the wake region, a large pocket of slow turbulent flow between $2<x/H<6$ for the $L/H=1$ case appears as a strong disturbance in the estimated pressure field.

**Fig. 2:**
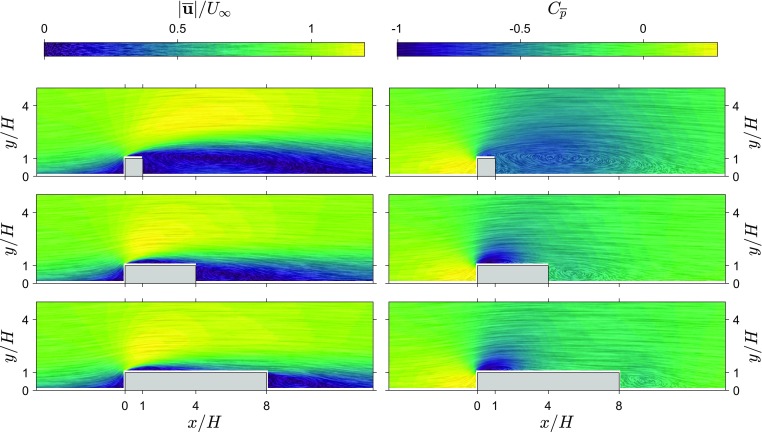
Average velocity magnitude field and pressure field from the 2D TH snapshot pressure approach for three rib lengths ($L/H=1,4,8$). Line integral convolution indicates local streamlines and strength. Left: average velocity magnitude field, $|\overline{\mathbf {u}}|/U_\infty$. Right: average pressure field, $C_{\overline{p}}$

### Pressure statistics from snapshot data

The instantaneous data from PIV snapshots can now be used to compute instantaneous pressure and its statistics (both mean and standard deviation) using the 2D TH approach. The statistics thus obtained can then be compared to the direct pressure transducer measurements (for both mean and standard deviation) as well as to the mean pressure estimate from the RANS method.

#### Average velocity and pressure

Figure 2 shows the mean velocity and mean pressure obtained from the 2D TH method (for three rib lengths, $L/H=1,4,8$). The velocity fields show that the separated flow region behind the obstacle decreases as rib length increases, and for the longer ribs a separation region near the leading corner splits from the wake region. The average pressure fields obtained using the 2D TH method show that the low pressure region near the leading corner becomes stronger with increasing rib length and for the longer ribs, there is a separate low pressure region in the wake of the rib.

**Fig. 3:**
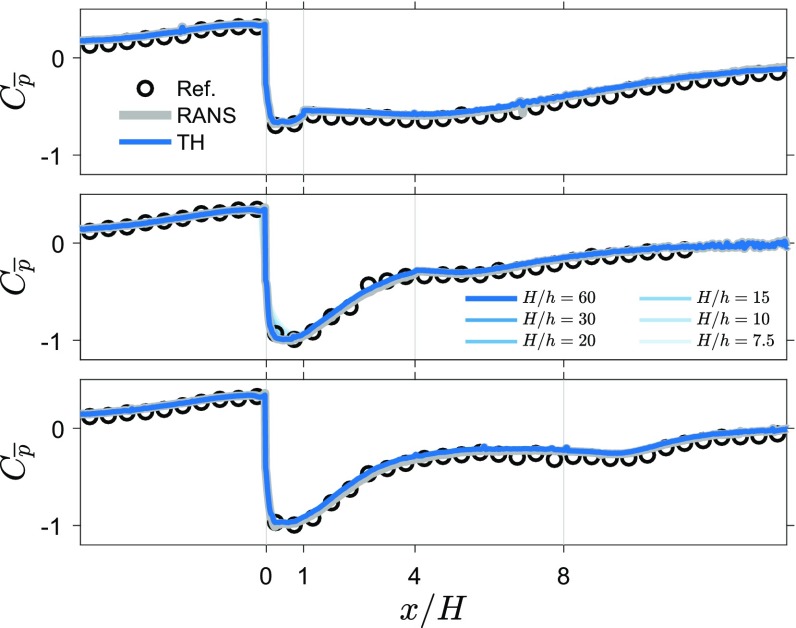
Average wall pressure using the 2D TH and RANS approach compared with reference measurements for three rib lengths ($L/H=1,4,8$), $C_{\overline{p}}$. Reduced resolution results are included for the $L/H=4$ case

**Table 1:** Average of absolute differences of mean pressure for RANS and 2D TH with reference pressure

Case	$\langle |\varDelta C_{\overline{p}}|\rangle _{\mathrm{RANS}}$	$\langle |\varDelta C_{\overline{p}}|\rangle _{\mathrm{TH}}$
$L/H=1$	0.043	0.047
$L/H=4$	0.024	0.029
$L/H=8$	0.033	0.042

**Fig. 4:**
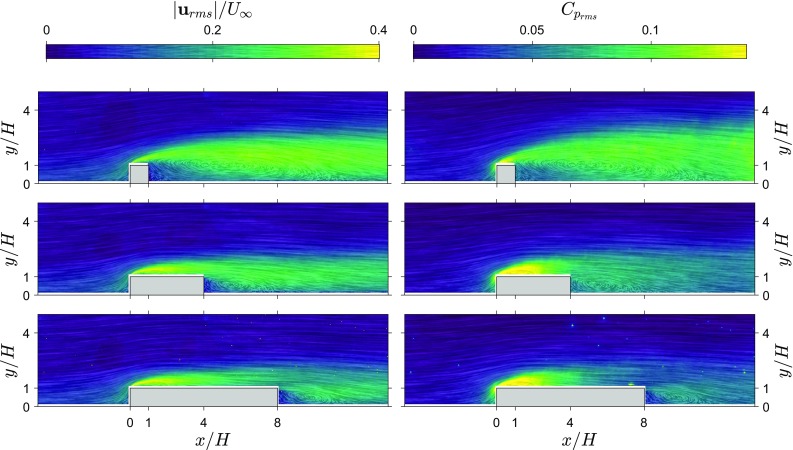
Fluctuating velocity magnitude field and pressure fluctuation field from the snapshot pressure approach for three rib lengths ($L/H=1,4,8$). Line integral convolution indicates local streamlines and strength. Left: RMS of the velocity fluctuations, $|\mathbf {u}_{\mathrm{rms}}|/U_\infty$. Right: RMS of the pressure fluctuations, $C_{\mathrm{p}_{\mathrm{rms}}}$

**Fig. 5:**
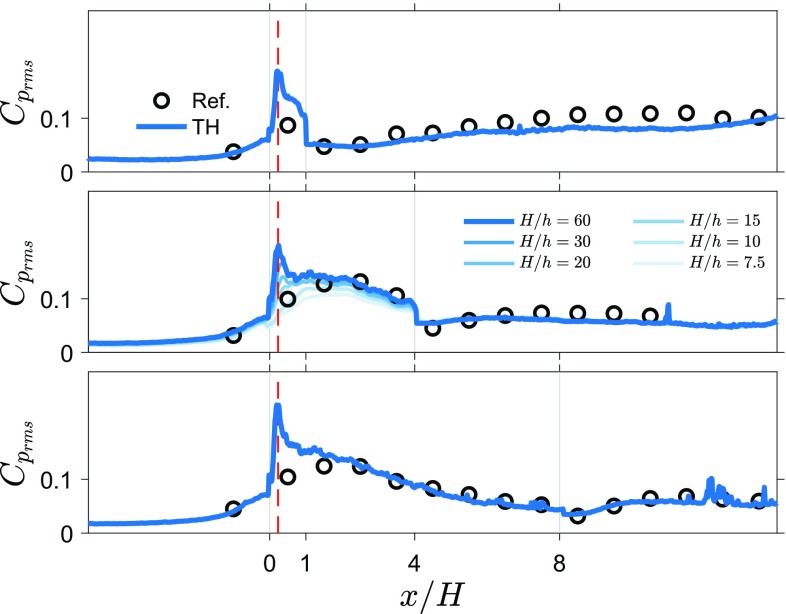
Fluctuating wall pressure ($C_{\mathrm{p}_{\mathrm{rms}}}$) from the 2D TH snapshot pressure approach compared with reference measurements for three rib lengths ($L/H=1,4,8$). Reduced resolution results are included for the $L/H=4$ case

Figure 3 shows line plots where the mean pressure from the 2D TH method is compared to the values obtained from the RANS method and to direct pressure measurements obtained at select locations at the wall. The line plots for the PIV estimate are obtained along the lowest wall-normal location where pressure could be determined. This is located approximately 3 mm from the surface. These comparative line plots show that the mean values from averaged instantaneous pressure estimates are close to the values obtained through the RANS approach. These line plots also indicate a good match between the direct measurements and estimates. To quantify the agreement, we determine the average absolute difference between them, i.e., $\langle |\varDelta C_{\overline{p}}|\rangle _{\mathrm{RANS}}=\langle |C_{\mathrm{p,RANS}}-C_{\mathrm{p,ref}}|\rangle$, where $|\cdot |$ denotes the absolute value and $\langle \cdot \rangle$ denotes average over all streamwise locations. Table 1 list the RMS residual for both techniques for all rib lengths. Overall, the 2D TH approach performs similar to the RANS approach with an average (over all cases) absolute difference of 0.039 and 0.033, respectively (which corresponds to $\approx 2$ Pa). The influence of reducing spatial resolution on the mean pressure results is minimal, and only minor attenuation of the mean pressure values near the leading corner can be observed.

#### Velocity and pressure fluctuations

**Table 2:** Absolute differences of fluctuating pressure from the 2D TH method with reference pressure and the ratio between the difference and the local reference value of fluctuating pressure

Case	$x/H=0.5$		$x/H\ne 0.5$	
	$|\varDelta C_{\mathrm{p}_{\mathrm{rms}}}|$	$\left| \frac{\varDelta C_{\mathrm{p}_{\mathrm{rms}}}}{C_{\mathrm{p}_{\mathrm{rms,ref}}}}\right|$	$\langle |\varDelta C_{\mathrm{p}_{\mathrm{rms}}}|\rangle$	$\left\langle \left| \frac{\varDelta C_{\mathrm{p}_{\mathrm{rms}}}}{C_{\mathrm{p}_{\mathrm{rms,ref}}}}\right| \right\rangle$
$L/H=1$	0.054	0.62	0.015	0.16
$L/H=4$	0.045	0.46	0.010	0.15
$L/H=8$	0.062	0.59	0.007	0.10

Results given for the worst case (near the leading corner, $x/H=0.5$) and average over all other locations ($x/H\ne 0.5$)

Fields of standard deviation of velocity and pressure obtained from the 2D TH method are shown in Fig. 4. Both the velocity fluctuations and pressure fluctuations have a similar distribution. High values of fluctuations start in the shear layer from the leading corner and spread out downstream. For the longer ribs, a region of locally higher fluctuations can be seen emanating from the trailing corner.

Figure 5 shows graphs of surface pressure estimates and measurements for three rib lengths. Standard deviation estimates appear to follow the directly measured values in most regions of the flow around ribs for all three cases. In the incoming boundary layer, the pressure estimates and direct measurements are similar. However, above the leading corner there is a big difference between the estimate and the reference (the peak value in pressure fluctuations is indicated with a red dashed line), indicating that this region proves difficult for the 2D TH approach. In the wake, for the shortest rib, there is a slightly better agreement than over the rib itself and the agreement between the 2D TH estimate and reference measurements improves with increasing rib length. Reducing the resolution attenuates the peak of pressure fluctuations near the leading corner, since the velocity fluctuations in the shear layer become under-resolved. Reducing the resolution to $H/h=20$ or lower (see results for $L/H=4$), also reduces the pressure fluctuation estimates along the whole top surface of the rib. This indicates that the spatial resolution of the time-resolved PIV measurements ($H/h=30$) was sufficient to capture the flow scales relevant for the pressure fluctuations.

To quantify the performance of estimating pressure fluctuations from snapshot PIV data, Table 2 provides absolute differences between the pressure fluctuations using the 2D TH approach and the reference pressure, $|\varDelta C_{\mathrm{p}_{\mathrm{rms}}}|=|C_{\mathrm{p}_{\mathrm{rms},\mathrm {TH}}}- C_{\mathrm{p}_{\mathrm{rms},\mathrm {ref.}}}|$, and the absolute of the ratio of the difference with the measured reference pressure, $|\varDelta \mathrm{p}_{\mathrm{rms}}/\mathrm{p}_{\mathrm{rms},\mathrm {ref.}}|$. The largest difference, located at the leading corner point, is provided and the average absolute difference excluding the leading corner point is given for all rib lengths. The maximum difference is $\varDelta C_{\mathrm{p}_{\mathrm{rms}}} \approx 0.06$ (which corresponds to $\approx 3$ Pa) for the leading corner, which results in an over prediction of the pressure fluctuations of about 60%. Since the leading corner point is obviously wrong and this is the region where TH is likely to fail completely (further discussion in Sect. 6.1), the average absolute difference for all locations excluding the leading corner is determined. This average absolute difference is $\langle |\varDelta C_{\mathrm{p}_{\mathrm{rms}}}|\rangle \approx 0.01$ (which corresponds to $\approx 0.5$ Pa), which results in predictions that are 10–15% off from the reference value.

## Pressure estimation from snapshot planar PIV applied to time-resolved planar PIV data: temporal correlation coefficient and comparison with time-resolved approaches

**Fig. 6:**
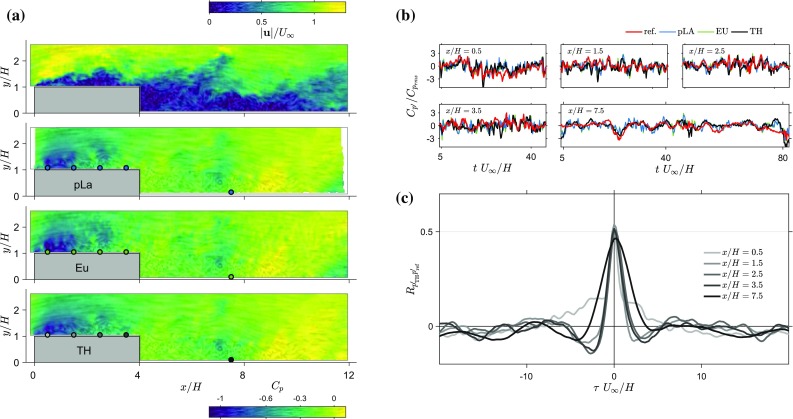
Time-series of snapshot pressure compared with time-resolved approaches. **a** Instantaneous velocity magnitude and pressure snapshots obtained with three different techniques from planar PIV. Line integral convolution indicates local streamlines and strength. An animation of the following 42 $H/U_\infty$ (125 ms) of flow is provided in the supplementary material. **b** Instantaneous time-series obtained from TR-PIV estimates, and the corresponding pressure transducer measurements covering the time interval of the animation ($tU_\infty /H=4$–46). **c** Cross-correlation between pressure estimates using the 2D TH method and the associated time-series for each transducer

To test the performance of the 2D TH approach beyond average and fluctuations, we apply it to time-resolved planar velocity data of the separated flow past a rib with length to height ratio of $L/H=4$ and compare the results with wall pressure time-series. Since these time-resolved data allow the application of 2D time-resolved approaches, we also include them for comparison.

### Experiment

To obtain the time evolution with simultaneous pressure measurements and PIV, we carried out an experiment in the “3x2” wind-tunnel at the University of Southampton where time-resolved planar PIV (TR-PIV) was used in conjunction with synchronised surface pressure transducers. A turbulent boundary layer was developed upstream ($\delta _{99} = 41$ mm) which impinged on a rib causing flow separation. The experiment was designed so that the rib height ($H=30$ mm) was comparable to the boundary layer thickness ($\delta /H = 1.37$), while the resulting span-wise aspect ratio ($L_{\mathrm{z}}/H=30$) ensured a statistically two-dimensional flow in the centre of the test section (Moss and Baker 1980; Kiya and Sasaki 1983). The length to height ratio was selected to be $L/H=4$. The free stream velocity was 10 m s$^{-1}$ and the resulting Reynolds number based on rib height was $Re_\mathrm{H}=$ 20,000.

The time-resolved data were obtained using three Phantom v641 high-speed cameras in conjunction with Sigma 105 mm f2.8 lenses and Nikon mount 1.4$\times$ Tele-converters. The cameras operated at 3200 Hz (resolution: $1536 \times 1028$ pixels) resulting in 1600 vector fields per second. A Litron LDY-304 high-speed laser was used to illuminate particles from a synthetic smoke machine at the same rate as the images. The image pairs were treated in Davis 8.3 with multiple correlation passes of interrogation regions decreasing in size from 64 $\times$ 64 to 24 $\times$ 24 pixels (with 50% overlap). The measured flow field extended from the leading edge of the rib, which is also the origin of the coordinate system, to 12*H* downstream of the leading edge and 2.5*H* above the flat-plate. Following Sciacchitano and Wieneke (2016), the uncertainty on the velocity fluctuations was approximately 2.5%. The final vector spacing was approximately 1 mm corresponding to $\approx 30$ vectors per rib height ($H/h=30$), which was sufficient to estimate the correct level of pressure fluctuations (see previous section).

At the same time as the velocity measurements, five Endevco 8507C-2 pressure transducers were used to record surface pressure fluctuations at $x/H=0.5,$ 1.5, 2.5, 3.5, and 7.5. To ensure synchronisation, the PIV trigger signal was also acquired by the pressure measurement system. A sixth Endevco 8510-1B was placed far upstream to acquire an uncorrelated pressure signal that could then be used for optimal noise cancellation as described by Naguib et al. (1996). These transducers were sampled at an acquisition rate of 25.6 kHz. The data were de-noised and low-pass filtered at 1280 Hz.

### Application of the 2D TH method to 2D measurements and comparison with 2D time-resolved approaches

To further establish the performance of the 2D TH method with planar measurements, time-series of estimated pressure are compared with wall pressure measurements. In addition, results of two time-resolved approaches are included for comparison. Figure 6 shows an example of a velocity snapshot and resulting pressure estimates, time-series of wall pressure for five locations for the different techniques and cross-correlation between wall pressure and the pressure from the 2D TH method.

Figure 6a contains sample snapshots of the instantaneous pressure estimate—using the three different methods: 2D EU, 2D pLA and 2D TH—around the rib. The evolution of streamlines is visualised with a line integral convolution as before. A video of the evolution of the velocity and pressure fields starting from the snapshots in Fig. 6a (using an technique similar to the dynamic LIC technique described by Sundquist 2003) is provided in the supplementary material. The three methods appear to produce similar results behind the leading edge, where shear layer roll-up and vortex shedding occurs. The range of pressure computed within the domain is comparable across all methods. However, The pLA and EU methods tend to introduce more fluctuations towards the edges of the domain of integration. In addition, the Eulerian method shows what seems to be a more noisy result throughout the integration domain. This is not a physical feature of the flow as highlighted in the following section.

The concurrent surface pressure fluctuation measurements are used to provide a reference for the pressure estimates described above. To compare these results, a pressure time-series is extracted from the nearest valid point to the pressure transducer. The measured and estimated time-series are synchronised in time therefore direct comparison is possible. Figure 6b depicts samples of the time-series obtained through direct measurements, and the estimates from 2D pLA, 2D EU and 2D TH methods for all transducer locations (as indicated in Fig. 6a). For the locations on top of the rib ($x/H=0.5$, 1.5, 2.5 and 3.5), the estimates appear to follow the reference signal quite well with no significant differences between the methods. In the wake region, the estimates follow the reference signal, but there is more differentiation between the methods. At higher frequencies, peaks in the pressure estimates from 2D EU and 2D pLA are present that are not mirrored in the reference signal. The 2D TH method seems to produce a smoother signal which follows the general trend of the two other estimates and also appears to follow the reference signal better.

To quantify the performance of the different approaches, cross-correlation coefficients between estimates and the direct measurements are determined. Figure 6c shows the cross-correlation $R_{p'_{\mathrm {TH}}p'_{\mathrm{ref}}}$ computed between the signal from five pressure transducers and the nearest pressure time-series estimated using the 2D TH method (location are also indicated in Fig. 6a). The figure shows the peak correlation is at zero time lag indicating that there is no phase difference between measured and estimated pressure at these locations. Figure 6c also shows that the peak correlation value decreases with increasing streamwise location. The same procedure was repeated for the two other estimation methods and no measurable time-delay was found between signals. The other methods also decrease in peak correlation value with downstream distance.

**Table 3:** Table containing the peak correlation coefficient between estimated and measured surface pressure

Location, $x/H=$	0.5	1.5	2.5	3.5	7.5
2D pLA	0.53	0.55	0.52	0.48	0.34
2D EU	0.57	0.57	0.52	0.48	0.34
2D TH	0.52	0.54	0.50	0.51	0.46

The estimates come from the nearest point to the surface with valid data, this may be several vectors above the surface due to reflections

The peak cross-correlation coefficient for all streamwise locations and all three methods is reported in Table 3. The 2D TH method shows a maximum $R_{p'_{\mathrm {TH}}p'_{\mathrm{ref}}}$ of 0.54 along the top surface of the obstacle, and 0.46 in the wake region. The highest correlation value is comparable to the other two methods. The comparable accuracy could be due to the fact that the TH method is more robust to measurement noise compared to the Eulerian and pseudo-Lagrangian methods (see de Kat and Ganapathisubramani 2013; Laskari et al. 2016). The 2D pLA and 2D EU methods exhibit a larger decrease in the peak correlation with downstream distance (the maximum difference is approximately 40%) than the 2D TH method estimates (the maximum difference less than 15%). This difference could be explained by the difference in sensitivity to measurement noise; two different sources of noise amplification exist in the pLA and EU methods (spatial and temporal gradients) while the TH method is only sensitive to errors in the spatial information. The wake transducer is located near the reattachment point ($x/H=7.5$ and $x/H\approx 8$, respectively) and the reference signal (see Fig. 6b) shows that the pressure changes in time are less sudden. This indicates that in that region the acceleration (temporal) information is likely less important (smaller accelerations) than the spatial information, and therefore, since it is not affected by temporal gradients, the TH method performs better.

## Pressure statistics in attached flows

The two assessments in this section consider attached wall-bounded flows. First, we use experimental data from a turbulent boundary layer where we compare the 2D TH method with its 3D counterpart and 3D time-resolved approaches, and second, we use numerical data from a turbulent channel flow to evaluate the performance and noise dependence.

The 2D TH method is applied to 2D snapshots of a turbulent boundary layer with $Re_\tau \approx 2300$ (for which time-resolved 3D velocity data are available, Laskari et al. 2016) to compare the pressure statistics of the 2D TH approach with different 3D approaches and DNS data. The applicability of the 2D TH approach is further evaluated using synthetic PIV data of a channel flow (Li et al. 2008; Perlman et al. 2007; Graham et al. 2013). Since the DNS pressure field is available, the performance of the method and its dependence on noise can be evaluated and used as a basis for the application of the method on other flows.

### Experimental assessment : turbulent boundary layer

The turbulent boundary layer experiment is described in Laskari et al. (2016) where multiple methods are compared for full 3D data. It was shown that the TH method applied to 3D information did not require time information and when compared to DNS outperformed the 3D EU approach, while it had a comparable accuracy with a 3D pLA approach. It should be noted that any Lagrangian approach will be accompanied by a significant volume loss due to convection. In line with the numerical assessment in the previous section, we used this dataset, in the limiting case of 2D velocity snapshots and we applied the 2D TH method on the individual streamwise–wall-normal planes of the original volumes, where only the *u* and *v* components of the velocity and their corresponding in-plane gradients were available.

For completeness of this work, a summary of the experiment is provided here and further details of the experiment can be found in Laskari et al. (2016): The turbulent boundary layer experiment was performed at the recirculating water channel (1.2 m $\times$ 0.8 m $\times$ 6.75 m) located at the University of Southampton Experimental Fluids Laboratory. The measurement location was about 5.5 m downstream of the contraction’s end where the flow was tripped. Each streamwise–wall-normal velocity plane was approximately 80 mm $\times$ 180 mm, in *x* and *y* with a digital resolution of 13 pixels/mm. A set of 3300 particle images was processed with Davis 8.2 using an iterative volume correlation with a final interrogation volume of $64 \times 64 \times 64$ pixels and an overlap factor of 75%. The nominal flow conditions, based on the 3300 evaluated vector fields, were $U_\infty \approx 0.66$ m/s, $\delta \approx 0.1$m, $Re_\tau \approx 2400$, and $Re_\theta \approx 5000$, while the resulting FOV was approximately $0.8\delta \times 2\delta$ in the streamwise and wall-normal direction, respectively. Pressure statistics were computed and for comparison with DNS data, the free stream RMS pressure was set to zero—analogous to the comparison approach by Tsuji et al. (2012).

**Fig. 7:**
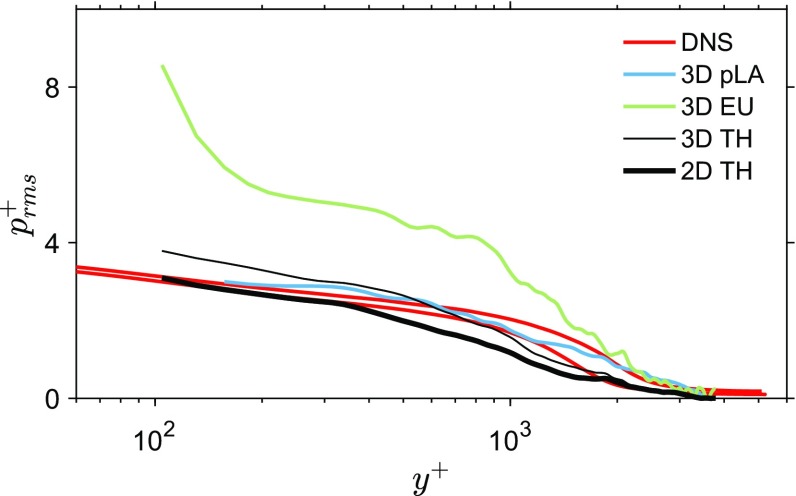
Pressure fluctuations from tomo-PIV in a turbulent boundary layer. Root-mean-square pressure, normalised with inner variables, using the 2D and 3D TH, 3D pLA, 3D EU methods ($l^+=104$, 3D EU and 3D pLA: $\mathrm{d}t^+=11.4$, 3D results from Laskari et al. 2016) and DNS results (Sillero et al. 2013, 2014; Borrell et al. 2013; Simens et al. 2009)

The RMS pressure values are in good agreement with DNS results of comparable Reynolds numbers (Sillero et al. 2013, 2014; Borrell et al. 2013; Simens et al. 2009). The estimated pressure statistics from the 2D TH method are compared with the 3D ones from Laskari et al. (2016) (Fig. 7). The 2D TH method shows comparable RMS pressure fluctuation values with both a 3D TH and a 3D pLA approach and all outperform the 3D EU approach.

### Numerical assessment : channel flow

For the numerical assessment of the 2D TH approach, we used the John’s Hopkins University Channel Flow Database (Li et al. 2008; Perlman et al. 2007; Graham et al. 2013), from which synthetic PIV volumes were constructed as described in Laskari et al. (2016). This time, similar to the boundary layer case in Sect. 5.1, instead of computing pressure using the 3D volumetric velocity fields, we extracted each individual streamwise–wall-normal plane of every volume to compute pressure using the 2D version of the governing equations (Eq. 11).

**Fig. 8:**
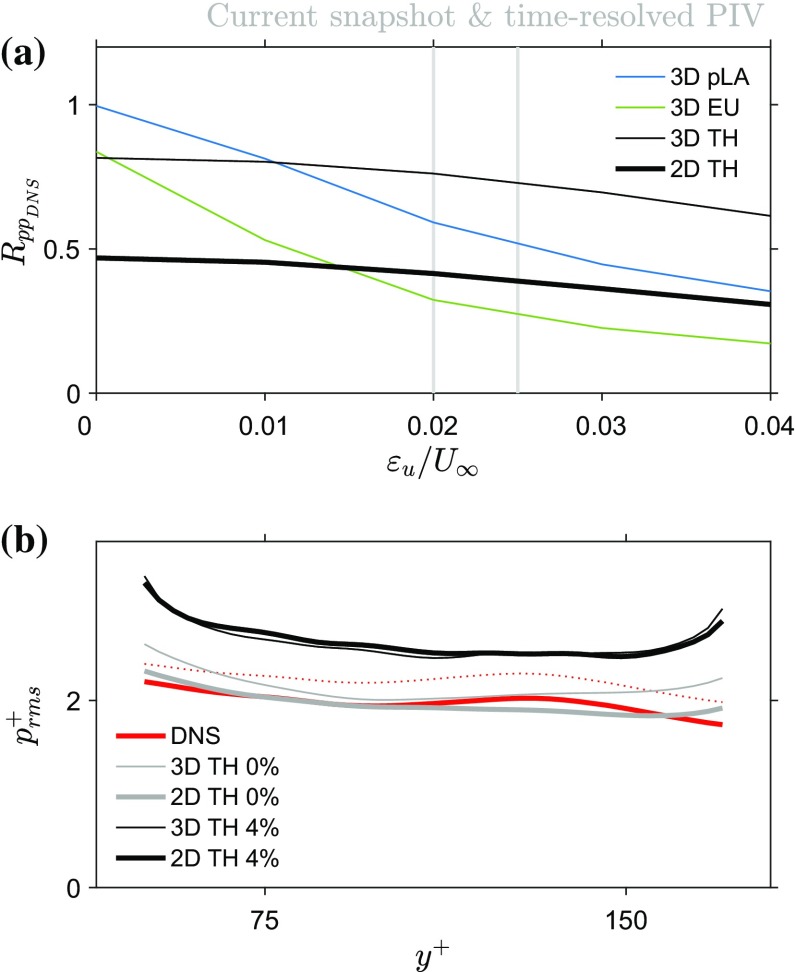
Pressure results from synthetic PIV on a channel flow. **a** Average correlation coefficient with varying noise for 2D and 3D results ($l^+=12$, 3D EU and 3D pLA: $\mathrm{d}t^+=1.28$, 3D results from Laskari et al. 2016). **b** Pressure fluctuations from synthetic PIV in a channel flow. Root-mean-square pressure, normalised with inner variables, using the 2D TH method together with the 3D TH method and DNS results (Li et al. 2008; Perlman et al. 2007; Graham et al. 2013). For completeness, DNS statistics without removing the volume average are indicated by the dotted red line

For the method’s assessment, and since the DNS pressure is available from the channel database, we use the correlation coefficient between the estimated and DNS pressure fields. Specifically, for each instantaneous volume selected (in total we used 30 volumes with sufficient time separation to be considered statistically independent, see also Laskari et al. 2016), we compute the correlation of the estimated 2D pressures in each streamwise–wall-normal plane of each volume with the corresponding 2D DNS pressure fields, and average over all planes and volumes. Results show that, in terms of instantaneous pressure fields, using 2D velocity data lead to an average correlation of 0.46 for low noise levels (marking a 40$\%$ drop from the 3D data case, Fig. 8a). Nevertheless, the method still shows robustness to noise influence and achieves better accuracy when compared to a 3D EU approach for the higher noise levels. Also, even though the loss of the third spatial dimension leads to a significant decrease in performance in the instantaneous fields, the pressure statistics are relatively unaltered in the case of zero noise and show only a small increase in the highest noise case ($\epsilon _u/U_{\mathrm{max}}=4\%$, Fig. 8b).

It should be noted here that, due to the small size and low number of available synthetic volumes when compared to the full database, the mean and RMS values of DNS pressure differ significantly from the converged database statistics (Li et al. 2008; Perlman et al. 2007; Graham et al. 2013). Also, for the estimated pressures, the average pressure on each volume is set to zero due to the lack of an appropriate Dirichlet boundary condition. Therefore, to be consistent in the comparison of DNS and estimated pressures, we effectively set the average DNS pressure of each volume to zero and compute the RMS pressures for both DNS and the TH method by averaging only over the number of data points available. The RMS pressure of the DNS is lowered by about 0.24, see Fig. 8b. This approach is similar to the removal of the mismatch in free stream turbulence as used by Tsuji et al. (2012) for comparing experiments and DNS.

## Discussion

Now that the performance of the 2D TH snapshot approach has been tested, we can compare its performance with another snapshot approach, the vortex-in-cell approach by Schneiders et al. (2016). In contrast with our approach, in which we simplify the experiment and analysis as much as possible, their approach uses full 3D information and a DNS-like data assimilation solver to estimate the pressure field.

First, we look at the correlation between the estimated and reference pressure from the numerical and time-resolved experimental tests. From our numerical tests, we found that the correlation between estimate and reference for the 2D approach is about 0.46, which matches well with the correlations found in our time-resolved analysis, 0.46–0.54. Based on our numerical tests, the correlation is expected to improve considerably when 3D data are available, by about 60% for the 2D to 3D TH approach, which suggests that a correlation of about 0.8 would attainable if we were to use 3D data, though this would come at the cost of complicating the experimental setup.

Schneiders et al. (2016) obtained pressure estimates in a smooth-wall turbulent boundary layer (an attached flow) to show that their vortex-in-cell approach together with snapshot 3D data and different boundary conditions results in a correlation coefficient of about 0.6 (the correlation was between the pressure estimate and wall-mounted microphones/pressure transducers). They compared their snapshot method with other time-resolved 3D data approaches. They found that a pseudo-Lagrangian method with a three-point stencil produces a correlation coefficient of 0.45 and one that uses a nine-point stencil produces a correlation coefficient of 0.65. This presumably indicates that a larger stencil, which will result in more smoothing in acceleration estimation, increases the correlation with reference values. Therefore, the range of correlation values obtained with a simple TH approach and 2D PIV data is within the range of (time-resolved) 3D methods and close (within 20%) to the value that an advanced data assimilation approach can attain using snapshot data. Note that the current 2D TH approach does not include any additional filtering of the velocity field (other than the those in determination of the vectors). This shows that reasonable (perhaps even good) pressure estimates can be made using a simple experimental setup and a simple approach to estimate the acceleration.

From our high-resolution 2D snapshot data, we found that the average wall pressure deviated less than 4% of the dynamic pressure from the reference wall pressure and was similar to the wall pressure from a RANS approach. Because in this study a separated flow is considered, there is variation in the average pressure field, in contrast with a smooth-wall turbulent boundary layer case, where the average pressure is of less interest (note that Schneiders et al. 2016 did not test the performance of their approaches in capturing the average pressure distribution on the wall).

On average the standard deviation of the pressure signal was within 10–15% of the reference pressure, see Table 2. Schneiders et al. (2016) did report the performance of their approaches in terms of pressure fluctuation ratios with respect to reference pressure measurements and found that the ratios, for their numerically intensive and advanced 3D technique to determine the pressure from 3D snapshot data, were within 11–12% (depending on the boundary condition that was employed to estimate the pressure). This shows that our simple 2D measurements with the 2D TH method perform as well as a more advanced snapshot approach based on a vortex-in-cell technique that needs 3D data. If one has access to 3D snapshot data, then van Gent et al. (2017) show that the 3D TH method can even outperform the approach of Schneiders et al. (2016).

The fact that the simple 2D planar PIV snapshot data in a separated flow in the current study provides good RMS pressure fluctuation estimates and moderate to good correlation with reference pressure suggests that the proposed method can be used in more challenging (predominantly 2D) flow conditions than purely convective flows (i.e. flows including significant separation) to obtain pressure statistics with reasonable fidelity. The estimates of mean and fluctuating pressure will come at minimal cost (both in terms of setup time, equipment and post-processing) in cases where use of snapshot planar PIV is already part of planned experiments (or enhance datasets already taken by extracting more information out of them).

Given the moderate to good agreement in RMS pressure fluctuations, the question rises on how close the instantaneous pressure estimates are to the true pressure. Therefore, the limitations of estimating instantaneous pressure will be discussed in the next section, before the limitations in estimating RMS pressure fluctuations are further discussed and a rule of thumb for the fidelity of estimated RMS pressure fluctuations will be provided.

### Limitations in estimating instantaneous pressure from snapshot planar PIV approach

One aspect that generally is ignored is the ability of a pressure estimation technique to accurately determine instantaneous pressure. Therefore, to assess the performance of the technique beyond the RMS and correlation statistics and estimate what the accuracy of the pressure in a single snapshot is, we will look at the RMS error, $\sigma _{\mathrm{err}}$, between the estimated and reference values and compare the current results with data from literature. First, we need to define how correlation, RMS ratio and RMS error are related. Following trivial algebra, we can obtain $\sigma _{\mathrm{err}}$ as a function of the estimated and reference RMS, and the correlation coefficient between the estimated and reference signal:

12 $$\begin{aligned} \frac{\sigma _{\mathrm{err}}}{\sigma _{\mathrm{ref}}}=\sqrt{1+\frac{\sigma _{\mathrm{est}}}{\sigma _{\mathrm{ref}}}\left( \frac{\sigma _{\mathrm{est}}}{\sigma _{\mathrm{ref}}}-2R_{\mathrm{est, ref}}\right) }. \end{aligned}$$

**Fig. 9:**
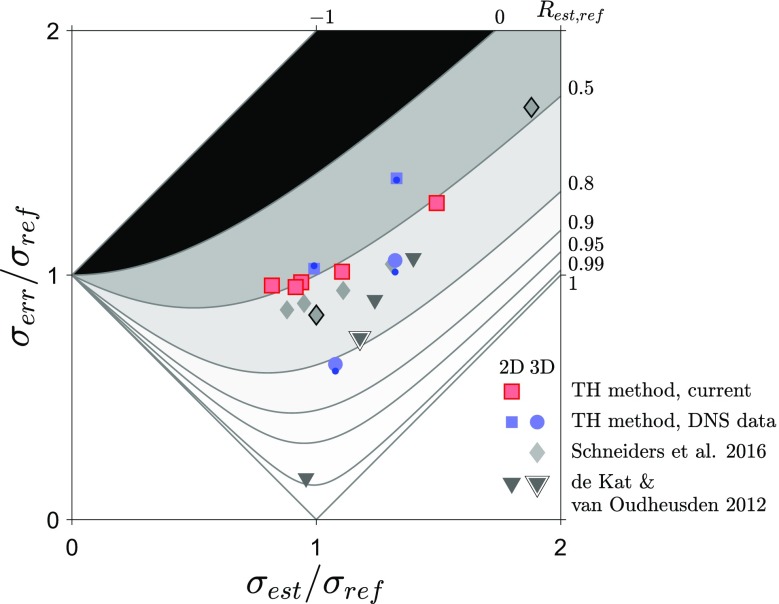
Ratio of the RMS error to the RMS of the (reference) signal, $\sigma _{\mathrm{err}}/\sigma _{\mathrm{ref}}$, of pressure fluctuations as a functions of ratio of RMS of the estimate to the RMS of the (reference) signal, $\sigma _{\mathrm{est}}/\sigma _{\mathrm{ref}}$. For the current data Eq. 12 is used with RMS ratio from snapshot PIV (Sect. 3) and correlation from time-resolved PIV data (Sect. 4). The accuracy of Eq. 12 is checked by computing the RMS error directly for the DNS data (Sect. 5.2) which are indicated by blue dots. For Schneiders et al. (2016), the time-resolved results have a black outline

From this equation, we can make a few observations. First, even if there is a perfect correlation (the shape of the signal is identical), the RMS error might not be zero due to a difference in gain. Second, if the estimate and the reference signal have the same RMS pressure fluctuations, then the correlation between them will determine what the RMS error between them will be. In short to have an RMS error that is less than 20% of the signal, one would need matching RMS and a correlation of at least 0.98. Unfortunately, such good matches in RMS combined with high correlation values are rarely reported for experiments.

A few works have provided estimates of RMS error for instantaneous pressure estimates using synthetic data assessments (see Charonko et al. 2010; de Kat and van Oudheusden 2012; van Gent et al. 2017), but do not relate them (directly) to the RMS signal and typically do not provide values for correlation between estimate and reference. However, using Eq. 12 we can now derive what the (relative) RMS error is from correlation values and RMS pressure fluctuation ratios. This allows us to use the experimentally determined values reported in de Kat and van Oudheusden (2012); Schneiders et al. (2016) and compare these with our current experimental (and synthetic) results.

In Fig. 9, relative RMS error values are plotted against the ratio of RMS estimate and reference, and the corresponding correlation values, $R_{\mathrm{est,ref}}$, are indicated by contours. The current results from the 2D TH method are compared with results from the 2D and 3D TH methods from the current synthetic data assessment and with the results from the two previous studies (de Kat and van Oudheusden 2012; Schneiders et al. 2016 if multiple estimates are available for the same approach, the lowest RMS error values are used) who provided RMS pressure fluctuation ratios and correlation values between the pressure estimates and reference. To check the effectiveness of Eq. 12, a direct evaluation of RMS error (blue dots in Fig. 9) is performed and found to be in good agreement with the values obtained using Eq. 12. From Fig. 9, it is evident that most results lie in the region where the RMS error is $>80\%$ of the RMS signal. Only one point has a low relative RMS error of 17% and this point is for the pressure on the side of a square cylinder using a 2D time-resolved approach (de Kat and van Oudheusden 2012). However, for this location the flow was predominantly 2D (de Kat and van Oudheusden 2012) and therefore is not representative for application of pressure estimates from PIV approaches in more complicated (3D) flows. The best (experimental) relative RMS error in 3D flow using 3D velocity data is in the wake of the square cylinder of de Kat and van Oudheusden (2012) which has a relative RMS error of 74%.

The current 2D TH method provided moderate to good estimates for the RMS pressure fluctuations, but as is clear from the RMS error results, see Fig. 9, the instantaneous pressure values for the 2D TH method should be treated with care because the relative RMS error is about 100%—similar to the other 2D approaches—and not far from the 3D snapshot and time-resolved approaches reported in Schneiders et al. (2016)—where the best result had 84% relative RMS error. In their synthetic assessment, van Gent et al. (2017) reported RMS errors for PIV based pressure estimation as low as 35% and for Lagrangian Particle Tracking based pressure estimation as low as 16%, both using noise free particle image data. The lowest RMS error for a snapshot based approach they reported was 56% for the 3D TH method, similar to our current RMS error estimate (64%). These results show that there is room for further improvements, but also indicate that the best instantaneous pressure estimates will most likely not have a RMS error lower than 16%.

Now that it is clear that pressure determination from PIV still requires some work to give good instantaneous pressure results, we will return to how well the current technique can estimate correct RMS pressure values. For the technique to be useful in practice, we will need to acknowledge its limitations and provide a rule of thumb based on measurement data so that an experimenter can determine the accuracy of the estimates.

### Limitations in estimating RMS pressure fluctuations from snapshot planar PIV approach

Now that we have established the performance and discussed the potential value of the (2D) TH method, it is wise to also consider its limitations and provide estimates for the applicability of the technique.

**Fig. 10:**
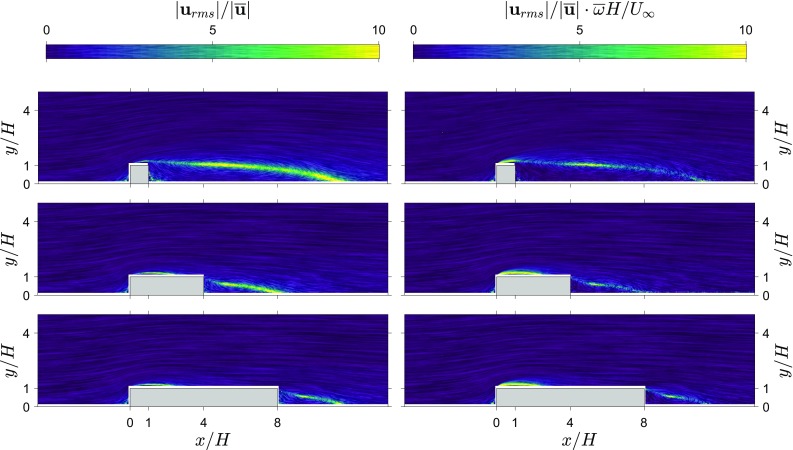
Ratio of velocity fluctuations to local mean velocity and this ratio multiplied with the local mean vorticity indicate where the pressure fluctuation estimation using the 2D TH method is limited. Results are shown for the three different rib lengths ($L/H=1,4,8$). Left: the ratio between velocity fluctuations and local mean velocity $|\mathbf {u}_{\mathrm{rms}}|/|\overline{\mathbf {u}}|$. Right: the ratio of the velocity fluctuation and the local mean velocity multiplied with the local mean vorticity $|\mathbf {u}_{rms}|/|\overline{\mathbf {u}}|\cdot \overline{\omega }H/U_\infty$

As discussed in the Sect. 3, the performance of the 2D TH method is worst near the leading corner of the flow. In the wake the differences are smaller, but still significant for the shortest rib. This is due to two main reasons. First, PIV as a technique has limitations in resolving the flow in shear layers due to high spatial gradients and issues raised by surface reflections. Also, the pressure estimates for the surface are taken from a plane with a small offset from the surface, inevitably leading to some discrepancies with the measured pressure values at the wall. The peak of the pressure fluctuations near the leading corner is located close to where the measurement plane intersects the shear layer. Within a turbulent shear layer one would expect the pressure fluctuations to be more severe due to roll-up of the shear layer into vortices. The second and perhaps more important reason for the discrepancy is related to the local validity of Taylor’s hypothesis. If the local mean velocity is low, the ratio between fluctuations and local mean velocity will become very large and thus invalidate the main assumption of small turbulent fluctuations being advected by the mean flow in Taylor’s hypothesis. Additionally, the shear layer emanating from the leading corner flaps up and down (see video) and this will cause large deviation in the advection velocity from the local mean velocity (and Lin 1953, showed that TH is further limited in shear flow).

In Fig. 10, the ratio of velocity fluctuations with the local mean velocity magnitude and this ratio multiplied with the local mean vorticity are shown. First, one can notice that the TH method is remarkably resilient to violation of the main assumptions involved in its definition (fluctuations small enough compared to the mean flow being advected by the local mean). In the wake region(s), the mean local velocity is very close to zero and the velocity fluctuations reach values in excess of ten times the local mean velocity. Despite this violation in the wake, the 2D TH method remains within a $\varDelta C_{\mathrm{p}_{\mathrm{rms}}}<0.025$. The wake results improved with increasing rib length (see Table 2) and if we look at the ratio between velocity fluctuations and local mean velocity (Fig. 10, left), we can see that the area where this ratio is large coincides with the deviation in pressure and the decrease in the region’s strength with increasing rib length.

The location with the largest difference in pressure fluctuations between the 2D TH method and direct measurements is on top of the ribs, but the value of the ratio between the velocity fluctuations and the local mean is not as large as in the wake. When we take the shear layer into account, see Fig.  10, right, we see that values on top of the rib get enhanced and the wake gets slightly attenuated. The peak values in $|\mathbf {u}_{\mathrm{rms}}|/|\overline{\mathbf {u}}|\cdot \overline{\omega }H/U_\infty$ appear near the leading corner, coinciding with the largest deviation in pressure fluctuations. The values in the wake are still coinciding with the deviations in pressure fluctuations in the wake and strength decrease with increasing rib length. This shows that we can use the mean vorticity and the velocity fluctuation ratio with the local mean velocity as an indicator for where the TH method will suffer. As a rule-of-thumb it appears that, when $|\mathbf {u}_{\mathrm{rms}}|/|\overline{\mathbf {u}}|\cdot \overline{\omega }H/U_\infty>10$ for a large region or at the boundary, the pressure fluctuations from the TH method start to suffer significantly—near leading corners in particular. However, in the other regions, good pressure fluctuation estimates can be obtained from the 2D TH snapshot pressure estimation approach.

## Conclusions

Pressure fields were extracted from multiple methods both in attached, and separated flows to evaluate the performance of Taylor’s hypothesis in estimating pressure from snapshot planar PIV measurements. Estimates of instantaneous pressure can be obtained with a correlation coefficient of about 0.5 with the Taylor’s hypothesis method applied to 2D data. As a case study, the mean pressure distribution in three separating and re-attaching flows was reproduced to within 4% of the dynamic pressure ($\approx 2$ Pa). Standard deviation of pressure was evaluated, and this exhibited good agreement with surface pressure transducer measurements, on average within 1% ($\approx 0.5$ Pa) of the dynamic pressure and 10–15% of the local pressure fluctuations. Determination and comparison of relative RMS error values indicate that instantaneous pressure estimates are not accurate (for the current approach and approaches from literature). Regardless, the RMS pressure estimates are in good agreement with reference measurements. The main limitation of the RMS pressure estimate (as expected) was the use of Taylor’s hypothesis in areas where there is strong shear-layer activity, leading to significant errors especially near the leading corner of the ribs considered here. As a rule-of-thumb it appears that, when $|\mathbf {u}_{\mathrm{rms}}|/|\overline{\mathbf {u}}|\cdot \overline{\omega }H/U_\infty>10$ for a large region or at the boundary, the pressure fluctuations from the TH method start to suffer significantly. Therefore, this study shows that it is possible to get reasonable estimates for full-field pressure from planar snapshot 2D PIV data and provides a rule-of-thumb on where the method is likely to perform well and where it falls short.

## Electronic supplementary material

Below is the link to the electronic supplementary material.

Supplementary material 1 (MP4 15 kb)

## Data Availability

Data published in this article are available from the University of Southampton repository at https://doi.org/10.5258/SOTON/D0773.

## References

[CR1] Bergeles G, Athanassiadis N (1983). The flow past a surface-mounted obstacle. J Fluids Eng Trans ASME.

[CR2] Borrell B, Sillero J, Jiménez J (2013). A code for direct numerical simulation of turbulent boundary layers at high Reynolds numbers in bg/p supercomputers. Comput Fluids.

[CR3] Cabral B, Leedom LC (1993) Imaging vector fields using line integral convolution. In: Proceedings of the 20th annual conference on computer graphics and interactive techniques. ACM, pp 263–270

[CR4] Charonko JJ, King CV, Smith BL, Vlachos PP (2010). Assessment of pressure field calculations from particle image velocimetry measurements. Meas Sci Technol.

[CR6] Davoust S, Jacquin L (2011). Taylor’s hypothesis convection velocities from mass conservation equation. Phys Fluids.

[CR7] de Kat R, Ganapathisubramani B (2013). Pressure from particle image velocimetry for convective flows: a Taylor’s hypothesis approach. Meas Sci Technol.

[CR8] de Kat R, van Oudheusden B (2012). Instantaneous planar pressure determination from PIV in turbulent flow. Exp Fluids.

[CR9] de Kat R, van Oudheusden B, Scarano F (2008) Instantaneous planar pressure field determination around a square-section cylinder based on time-resolved stereo-PIV. In: In Proc. of the 14th Int symp on applications of laser techniques to fluid mechanics, Lisbon

[CR10] Favre A, Gaviglio J, Dumas R (1955) Some measurements of time and space correlation in wind tunnel. Technical Report NACA-TM-1370. National Advisory Committee for Aeronautics

[CR11] Fisher M, Davies P (1964). Correlation measurements in a non-frozen pattern of turbulence. J Fluid Mech.

[CR12] Fujisawa N, Tanahashi S, Srinivas K (2005). Evaluation of pressure field and fluid forces on a circular cylinder with and without rotational oscillation using velocity data from PIV measurement. Meas Sci Technol.

[CR13] Geng C, He G, Wang Y, Xu C, Lozano-Durán A, Wallace J (2015). Taylor’s hypothesis in turbulent channel flow considered using a transport equation analysis. Phys Fluids.

[CR14] Ghaemi S, Ragni D, Scarano F (2012). PIV-based pressure fluctuations in the turbulent boundary layer. Exp Fluids.

[CR15] Graham J, Lee M, Malaya N, Moser R, Eyink G, Meneveau C, Kanov K, Burns R, Szalay A (2013) Turbulent channel data set. http://turbulence.pha.jhu.edu/docs/readme-channel.pdf

[CR16] Gurka R, Liberzon A, Hefetz D, Rubinstein D, Shavit U (1999) Computation of pressure distribution using PIV velocity data. In: Workshop on particle image velocimetry, vol 2

[CR17] Hosokawa S, Moriyama S, Tomiyama A, Takada N (2003). PIV measurement of pressure distributions about single bubbles measurement of pressure distributions about single bubbles. J Nucl Sci Technol.

[CR18] Jakobsen ML, Dewhirst TP, Greated CA (1997). Particle image velocimetry for predictions of acceleration fields and force within fluid flows. Meas Sci Technol.

[CR19] Kim J, Hussain F (1993). Propagation velocity of perturbations in turbulent channel flow. Phys Fluids.

[CR20] Kiu MH, Banks DC (1996) Multi-frequency noise for LIC. In: Proceedings of the 7th conference on visualization’96. IEEE Computer Society Press, pp 121–126

[CR21] Kiya M, Sasaki K (1983). Structure of a turbulent separation bubble. J Fluid Mech.

[CR22] Laskari A, de Kat R, Ganapathisubramani B (2016). Full-field pressure from snapshot and time-resolved volumetric PIV. Exp Fluids.

[CR23] Li Y, Perlman E, Wan M, Yang Y, Meneveau C, Burns R, Chen S, Szalay A, Eyink G (2008). A public turbulence database cluster and applications to study lagrangian evolution of velocity increments in turbulence. J Turbul.

[CR24] Lin C (1953). On taylor’s hypothesis and the acceleration terms in the Navier–Stokes equations. Q Appl Math.

[CR25] Liu X, Katz J (2006). Instantaneous pressure and material acceleration measurements using a four-exposure PIV system. Exp Fluids.

[CR26] Longmire E, Ganapathisubramani B, Marusic I, Urness T, Interrante V (2003). Effective visualization of stereo particle image velocimetry vector fields of a turbulent boundary layer. J Turbul.

[CR27] McClure J, Yarusevych S (2017). Optimization of planar PIV-based pressure estimates in laminar and turbulent wakes. Exp Fluids.

[CR28] Moss D, Baker S (1980). Re-circulating flows associated with two-dimensional steps. Aeronaut Q.

[CR29] Murai Y, Nakada T, Suzuki T, Yamamoto F (2007). Particle tracking velocimetry applied to estimate the pressure field around a Savonius turbine. Meas Sci Technol.

[CR30] Naguib A, Gravante S, Wark C (1996). Extraction of turbulent wall-pressure time-series using an optimal filtering scheme. Exp Fluids.

[CR31] Perlman E, Burns R, Li Y, Meneveau C (2007) Data exploration of turbulence simulations using a database cluster. In: Proceedings of the 2007 ACM/IEEE in supercomputing, pp 1–11

[CR32] Phillips N, Knowles K, Bomphrey RJ (2015). The effect of aspect ratio on the leading-edge vortex over an insect-like flapping wing. Bioinspir Biomim.

[CR33] Scarano F (2013). Tomographic PIV: principles and practice. Meas Sci Technol.

[CR5] Schanz D, Gesemann S, Schröder A (2016). Shake-the-box: Lagrangian particle tracking at high particle image densities. Exp Fluids.

[CR34] Schneiders JFG, Pröbsting S, Dwight RP, van Oudheusden BW, Scarano F (2016). Pressure estimation from single-snapshot tomographic PIV in a turbulent boundary layer. Exp Fluids.

[CR35] Schröder A, Schanz D, Michaelis D, Cierpka C, Scharnowski S, Kahler C (2015). Advances of PIV and 4D-PTV ’Shake-the-box’ for turbulent flow analysis -the flow over periodic hills. Flow Turbul Combust.

[CR36] Sciacchitano A, Wieneke B (2016). PIV uncertainty propagation. Exp Fluids.

[CR37] Sillero J, Jiménez J, Moser R (2013). One-point statistics for turbulent wall-bounded flows at Reynolds numbers up to $$\delta ^+$$$$\approx $$ 2000. Phys Fluids.

[CR38] Sillero J, Jiménez J, Moser R (2014). Two-point statistics for turbulent boundary layers and channels at Reynolds numbers up to $$\delta ^+$$$$\approx $$ 2000. Phys Fluids.

[CR39] Simens MP, Jiménez J, Hoyas S, Mizuno Y (2009). A high-resolution code for turbulent boundary layers. J Comput Phys.

[CR40] Sundquist A (2003). Dynamic line integral convolution for visualizing streamline evolution. IEEE Trans Vis Comput Graph.

[CR41] Taylor GI (1938). The spectrum of turbulence. Proc R Soc Lond A Math Phys Sci.

[CR42] Tsuji Y, Imayama S, Schlatter P, Alfredsson PH, Johansson AV, Marusic I, Hutchins N, Monty J (2012). Pressure fluctuation in high-Reynolds-number turbulent boundary layer: results from experiments and DNS. J Turbul.

[CR43] Van der Kindere J, Ganapathisubramani B (2018). Effect of length of two-dimensional obstacles on characteristics of separation and reattachment. J Wind Eng Ind Aerodyn.

[CR44] van Gent P, Michaelis D, van Oudheusden B, Weiss PE, de Kat R, Laskari A, Jeon Y, David L, Schanz D, Huhn F, Gesemann S, Novara M, McPhaden C, Neeteson N, Rival DE, Schneiders JFG, Schrijer F (2017). Comparative assessment of pressure field reconstructions from particle image velocimetry measurements and Lagrangian particle tracking. Exp Fluids.

[CR45] van Oudheusden B (2013). PIV-based pressure measurement. Meas Sci Technol.

[CR46] van Oudheusden B, Scarano F, Roosenboom E, Casimiri E, Souverein L (2007). Evaluation of integral forces and pressure fields from planar velocimetry data for incompressible and compressible flows. Exp Fluids.

[CR47] Violato D, Moore P, Scarano F (2011). Lagrangian and Eulerian pressure field evaluation of rod-airfoil flow from time-resolved tomographic PIV. Exp Fluids.

[CR48] Zaman K, Hussain A (1981). Taylor hypothesis and large-scale coherent structures. J Fluid Mech.

